# Feature-Based Framework for Inspection Process Planning

**DOI:** 10.3390/ma11091504

**Published:** 2018-08-22

**Authors:** Fernando Romero Subirón, Pedro Rosado Castellano, Gracia M. Bruscas Bellido, Sergio Benavent Nácher

**Affiliations:** Department of Industrial Systems Engineering and Design, Jaume I University, 12071 Castellón de la Plana, Spain; fromero@uji.es (F.R.S.); rosado@uji.es (P.R.C.); benavens@uji.es (S.B.N.)

**Keywords:** feature-based modeling, inspection planning, dimensional and geometrical specification, process specification, collaborative and integrated product-process development

## Abstract

Feature-based approaches have been profusely used in the last decades to incorporate domain-specific knowledge in the design and development of technical systems that, according to the new Concurrent Engineering approaches, involves not only the definition of the product, but also of the required manufacturing/inspection/assembly process and the corresponding production system. Although the ability of feature-based modeling to ease and integrate knowledge intensive processes has always been recognised, in practise the different feature-based modeling proposals are strongly dependent on the domain and on the development stage of the solution (conceptual, detailed, etc.). On the other hand, inspection process planning, including the design and selection of the technical system to realize the dimensional and geometrical verification of the manufactured artefacts, has been traditionally considered separately from the rest of the manufacturing process planning, and even also from the product functional specification tasks. In this work, a feature-based framework for inspection process planning, based on a similar approach to the one applied in GD&T (Geometrical Dimensioning & Tolerancing) specification, analysis and validation of product artefacts, is presented. With this work, the proposed framework and feature concept ability to model interaction components belonging to both the product and the inspection system (inspection solution) is proved. Moreover, to facilitate the Collaborative and Integrated Development of Product-Process-Resource, the Inspection Feature has been conceived as a specialization of a generic Feature previously proposed by the authors.

## 1. Introduction

To face nowadays intense global competition, companies require manufacturing systems to be more flexible, adaptable, reactive and interoperable. This circumstance, together with the development of new information and communication technologies, such as Service-Oriented Computing (SOC)/Service-Oriented Architecture (SOA) or Cloud Computing and Web Services, has given rise to the emergence of several manufacturing technical and operational paradigms such as Digital Manufacturing, Reconfigurable Manufacturing, Service Manufacturing or Cloud Manufacturing, among others [1,2,3,4,5].

**Figure 1:**
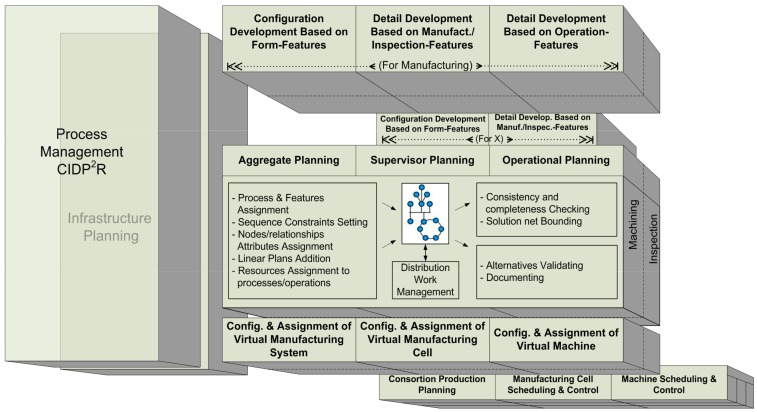
Functional architecture of the Collaborative and Integrated Development of Product, Process and Resource (CIDP^2^R) process [8].

To be able to cope with the above-mentioned characteristics and to encourage collaboration in complex current manufacturing systems, a more reactive, adaptable and distributed Process Planning with an enhanced connection to production scheduling and product design is required [6,7,8]. The need that Process Planning has these characteristics and that is configured as a central element in an integrated product-process-resource system, was already stated many years ago, from one of the first proposals by the authors of [7] up to the proposal of a framework for the Collaborative and Integrated Development of Product, Process and Resource (CIDP^2^R) process [8,9]. In order to reach high levels of integration and adaptability, the proposal by the authors of [8,9] is based on a service-oriented architecture and locates Process Planning as a central activity interacting with Design and Production Planning and Control activities. In addition, this last proposal fosters an approach based on (central part of Figure 1): (a) a unified activity model valid for any process planning activity; and (b) a unified product-process-resource information model. This information model is based on a feature concept able to consistently support the development of solutions to meet functional requirements in the different involved domains (product design, manufacturing process planning, inspection process planning, etc.) at any abstraction/specialization level.

These feature-based approaches are also the basis of recent works in the newest and current Cloud-based Design and Manufacturing contexts. The need of a feature-based approach together with service-oriented architectures for data exchange in Cloud-Based Design and Manufacture contexts is stated in [10]. Similarly, cloud and feature-based Functional Blocks (FB) technologies to develop a Cloud Distributed Process Planning system that works as a central service aimed at increasing responsiveness and adaptability in current collaborative environments is adopted in [3]. However, all these proposals make use of a very specific feature concept and highlight the need of a generic feature concept able to support frameworks such as the previously mentioned CIDP^2^R one.

The unified activity model developed in the CIDP^2^R (Figure 1) considers the integration of the activities and their relationships in two dimensions [11]. One of the dimensions refers to the development process maturity and distinguishes three levels: aggregate, supervisory and operational. The second dimension refers to the perspective and takes into account the product, the needed process plans and the required resources. In [11], the supervisory level and process planning activities are thoroughly described, and particularly the manufacturing and inspection processes integrationin order to encourage the use of new in line inspection (in and post process) capabilities, especially on-machine measurement, to obtain even real-time performance information and improve system reactivity. This has been in increasing need in recent years, due to the appearance of hybrid machines that combine processing technologies (e.g., subtractive and additive manufacturing) with new measurement technologies.

The requirement of a generic feature concept to support the CIDP^2^R process, led to the proposal of the Unified Application Feature (UAF) framework, which includes the definition of a generic Application Feature, and which will be briefly reviewed in the next section [12]. In addition, a Specification Feature, such as a specialization of the Application Feature, was proposed in [13]. This Specification Feature considers geometry with defects to support all the activities of the CIDP^2^R process where the consistent representation and treatment of dimensional and geometrical variations is essential: product specification, process (manufacturing and inspection) specification, and resource assignment. Additionally, in the same work, a system-oriented and tolerance-driven artefact model was also defined, where the workpiece is understood as a part of an assembly (assembly model), valid for all the product life cycle phases (final product assembly, machining process assembly, inspection process assembly), which is required to achieve unification.

According to the above, and in addition to the definition of a specific feature for the inspection domain, this work aims to prove that the UAF framework, based on the proposed Application Feature, has the sufficient generality to provide the required flexibility in order to define feature specializations. These Application Feature specializations are not only in the product domain, but also in the process planning and resource assignment and configuration domains.

The rest of this paper is organised as follows. In Section 2, a generic specification feature model developed by the authors in previous work is briefly summarized. Section 3 presents the proposal of an Inspection Feature, as a subtype of Specification Feature, which enables Supervisory Inspection Plan specification and validation based on the inspection assembly, resulting from the assembly of the subject part for inspection and the measurement resource (including fixture, probe, equipment, etc.). Finally, Section 4 concludes with a summary of the main contributions and indicates some future work.

## 2. Background and Methodology

Traditionally, the dimensional and geometrical specification exercises are carried on the assembly corresponding to the final product and their objective is to establish and validate product functionality through the geometric specification of all its individual parts. However, along the different product lifecycle stages, each of these parts participates in other assemblies required for its realization (manufacturing and inspection). These process assemblies (manufacturing and inspection) that are established for process plan specification, analysis and validation (inspection blueprints), in addition to the part, include the resources on which the part is fixtured in the different process set-ups. Therefore, a feature-based framework for specification is necessary to enable, in a dual and consistent manner, a uniform product and process plan specification considering, analyzing and validating two different types of assemblies: product and process assemblies (manufacturing and inspection).

Before presenting the Feature-based Framework for Geometric Specification, in the first part of this section, a general review of feature concept and feature-based modeling frameworks is carried out. One of the generic featured-based frameworks used for geometric specification and aimed at fulfilling the requirements of a consistent product and process plan specification, is summarized in the second part of this section. This framework has been presented in prior published authors’ work [12,13]. The third part of this section presents the parts of the framework for geometric specification that includes a geometry model, a specification feature model and an assembly model. Finally, the section ends with a summary of the methodology used to develop and validate the proposal of an Inspection Feature.

### 2.1. Literature Review on Features Definitions and Modeling Frameworks

The feature is a concept that was incorporated in the design and development of technical systems by the end of the last century, especially in the Computer Aided Design and software product line engineering fields. In the first one, a feature represents the engineering meaning of the geometry of a part or assembly [14]. In the second one, feature modeling is a common approach to manage variability supporting the establishment of a product line configuration that meets multiple, and often contradictory, requirements [15]. However, in recent decades this approach from the software domain was progressively applied to the management of technical systems, and particularly to mechatronics systems [16]. This fact has been fostered by the customised mass production paradigm, since feature modeling offers a transparency for capturing and visualizing optional and alternative conceptual design solutions that the traditional requirement specification process does not provide [17].

If the feature concept and definition in specialised literature is analyzed, it can be noted that the feature usually depends on the context of the application domain and that, additionally, the concepts used remain still ambiguous and very often contradictory, even when the domain context is perfectly established. The consistency of the meaning given to the feature in different engineering areas was analyzed in [18], concluding that the feature concept has been employed with very different representation purposes such as an abstract concept, a set of properties, the material it is constituted of, a component structure, etc. Although the authors of [18] reveal this reality, the reason for it is not explained. From this paper author’s perspective, the reason for this reality is that during the design exercise (intent) the engineer needs the support of different entities, although they should be unified to ensure consistency.

In addition, feature generic definitions and frameworks aimed at unifying the concept and the development of feature-based models and to support the interoperability among the applications can be found in all domains, from the most specific to the most general ones. A very general definition describes the feature like *anything about the thing being designed that’s from interest* [19]. Based on this definition [19] establishes three types of feature: *Functional, Behavioural and Structural Feature*. Other authors define more specifically the feature as: (1) *An information object (feature type), always related to an artefact, that specifies engineering intent* [18]; (2) *A property that is relevant to some stakeholders and is used to discriminate between concept instances.* In the case of technical systems, these properties can be structural (e.g., shape, size), behavioural (e.g., an operation mode) and functional (e.g., cruise control of a car) [20]; or (3) *As abstractions or groupings of requirements describing structural, behavioural or functional properties of a system that are relevant and understandable for different stakeholders* [21].

Considering all the previous definitions, it can be concluded that the feature must always be understood as something that facilitates the specification and therefore, in order to do so, it must be perceived as an informational object that belongs to the design solutions space. In this design solutions space, two different sub-spaces can be distinguished: the design (functional) rationale one and the design (structural) components one. Both sub-spaces can be established at different abstraction levels. The feature is able to describe solutions either in one of them or in both (mapping the functional solution with the structural solution).

Moreover, instanced feature objects must always be described in a simplified way (label), according to [17] by a single word or a short line of text. This last circumstance and the fact that the feature is always related with the function (Functional Feature), with the technical product (Structural Feature), or with both, is the reason why the feature is frequently mistaken with a function and/or with a product. A car, a car impeller or a car body are instances of (functional) features at different conceptual levels present in the feature (tree) model representing the design intent (rationale) analysis. Likewise, an engine, a piston or a rod are also examples belonging to more specific analysis levels and that are linked with their embodiment, facilitating the relation with the components and their (structural) features, which represent the virtual product structure.

According to [22], the engineering community lacks a common way to represent features, which is suitable to support data sharing and interoperability between systems and communities. Two feature frameworks, both using Unified Modeling Language (UML) class diagrams, are highlighted by [22]: the Generic Feature proposed by [23] and the Unified Application Feature proposed by [12]. The Unified Application Feature (UAF) is the basis for this work and, therefore, it will be briefly described in the next section.

### 2.2. Unified Application Feature Framework

The UAF framework is based on the essential need that the feature encapsulates the design intent, as proposed by de PPO (Product-Process-Organization)model [24,25], and is enhanced with ideas from feature modeling techniques in software domain, such as FODA (Feature-Oriented Domain Analysis), and particularly, with the approach proposed by [15] who considers different types of feature. The UAF framework has been represented using UML class diagrams. The choice of UML as representing language is due to two main reasons. Firstly, UML is normally used in many engineering domains to describe a data model. Secondly, UML conceptual descriptions translate well to OWL, which is a language commonly employed to develop ontologies.

This framework defines a generic feature, the Application Feature [12,13], as a container of different feature categories, likewise other authors had already done [15,16]. The Application Feature is able to support any design solution, including the mapped functional and structural design solutions and independently of the abstraction/specialization level.

**Figure 2:**
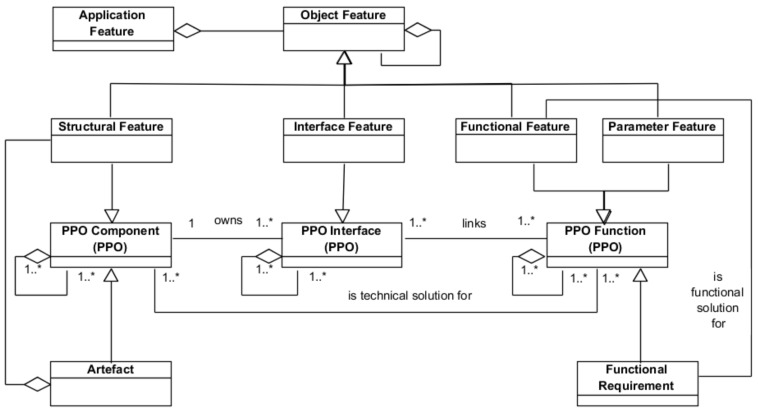
Updated basic structure of the Application Feature Model and its relation with the Product-Process-Organization (PPO) model.

The proposed Application Feature, which is described in Figure 2 using a UML class diagram, is defined as an aggregation of other features (Object Feature). An Object Feature, which represents a valuable aspect for the stakeholders, is an information entity of one of the following four categories: (a) *Functional Feature*, which represents the way in which the artefact may interact with other systems. Therefore, it represents a functional solution for a functional requirement (resulting from, for example, product or process specification); (b) *Interface Feature*, which represents the artefact elements that play a port role in the interaction with other systems; (c) *Structural Feature*, which represents the artefact configuration, that is, the artefact components and their structural relationships (e.g., part of, composed of, formed by, etc.); and (d) *Parameter Feature*, which contains all other non-functional properties with required quantification by values or quality assignment (e.g., colour, weight, volume, etc.).

Figure 2 also illustrates that the four Object Features are specializations of the three fundamental classes (*Component*, *Interface* and *Function*) of the PPO model. In this way, the fundamental classes inherit the properties of the PPO, easing collaborative work [24,25]. Additionally, the labels of the association relations established between the PPO classes determine the relationships typical of an engineering process driven by the design intent.

In contrast to [23], the UAF model also supports the feature-based description of the design solutions and, as can be seen in Figure 2, includes the relationship with the components hosting the features (*Artefact*) and the relationship with the functions (*Functional Requirement*) fulfilled by the features. In this sense, the proposed definition of the Application Feature is also in line with the concepts behind of the so-called Configurable Components used in product platform design that are understood as autonomous knowledge-carrying configurable generic subsystems [26]. Another shared characteristic of the Application Feature with the Configurable Components is that they allow component links through their interface and interaction elements. Thus, the Application Feature is an information object that enables product and manufacturing system (technical artefact) design based on the definition of the corresponding platforms. In addition, similarly to the Configurable Components, the Application Feature makes no distinction between the product artefact and the manufacturing system artefact, considering both in the same way (technical artefact). This allows for the establishment of interrelationships between product and manufacturing system Application Features by means of an Interaction Feature relating Interface Features of the Product and the Manufacturing System.

### 2.3. Feature-Based Framework for Geometric Specification

As has been mentioned, the geometric specification exercises are carried out on assemblies (product or process). These assemblies, which represent technical solutions for product or process functional requirements, are made of parts interacting through their geometric interfaces. The representation of these geometric interfaces for the specification exercise requires of appropriate geometry models in order to consider and limit the geometric variability. These geometry models are described in the first part of this section. The proposed geometry models are used in the Specification Feature Model presented in the second part of this section. Finally, the Assembly Model, which enables the establishment of a chain for each functional requirement, is presented.

#### 2.3.1. Geometry Model for Specification

During the product specification task, the designer works with imaginary geometries with defects of the parts of the product assembly. Based on these imaginary geometries and considering the geometrical conditions of the final product function, the designer carries out several simulation exercises on the product final assembly, with the aim of specifying permissible geometric deviations (tolerances) for each individual part of the assembly. Likewise, during the inspection process specification task, the planner works with imaginary geometries of the components of the measurement assemblies, devised solutions to measure the subject part of the inspection, and carries out different simulation exercises in order to specify the permissible uncertainties for the planned measurement assembly. In this way the appropriateness of the measurement assembly solutions (reference surfaces, fixture, probes, etc.) established to determine the GD&T (Geometrical Dimensioning & Tolerancing) characteristics specified for the subject part of the inspection is validated. The types of supported geometric defects need to be compatible with the selected simulation tool used and with the type of deviation that the measurement instrument or equipment is capable of extracting. Alike simulation exercises are also present in the specification of any manufacturing process, such as the machining process, with the aim of specifying the manufacturing systems (dimensional and geometrical) capabilities.

From all the above-mentioned, it can be gathered that process (manufacturing or inspection) specification exercises, similar to what the GPS (Geometrical Product Specification) standard establishes for product specification and its verification, are also based on the distinction between the real world, where several and different realizations of the part exist, and the imaginary models (surface models), used to represent those realizations. The GPS standard establishes a similar distinction for product specification and its verification [27] defining three types of surface models (nominal, skin and extracted).

**Figure 3:**
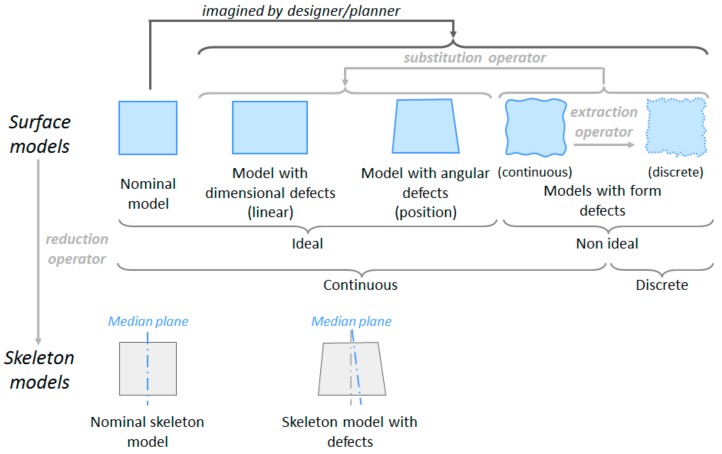
Geometry models for product/process specification.

Figure 3 shows the digital models with defects considered in this work that can be used in the different simulation tools and that are linked to the conceptual skin model. Two types of these surface models are considered: ideal models, which are defined by a parametrized equation, and non-ideal models, which can be defined by a set of surface patches (continuous) or by a set of points, segments or tessellation elements (discrete). In practice, it is unfeasible to obtain the non-ideal continuous model, since it would require a large amount of complex information. Therefore, discrete models, which are obtained by sampling on the real part, are used in the specification exercise. This discrete model is the one considered by GPS, and hence always assuming a measurement method based on discrete digitalization. The non-ideal models can be simplified to different ideal models. If the simplification process neglects the form and orientation defects of the surfaces, models with dimensional (linear) defects and models with angular (position) defects are obtained respectively. The simplification process can lead to skeleton models with defects, when the geometries participating in the functional condition of the assembly are geometric elements derived from surface elements. If the simplification process is applied to ideal models, substitution and/or reduction operators are involved. Extraction operators are the ones involved when simplifying from a non-ideal continuous model to a discrete model.

Although in recent literature discrete models to represent the geometry with defects have been proposed [28], the majority of the analysis methodologies and tools use ideal continuous geometries and geometric tolerancing models based on variational geometries that do not include form defects (Degree of Freedom–D.o.f., Small Displacement Torsor–SDT, etc.). Therefore, if the analysis is carried out using one of these techniques, the representation of the geometry with defects (skin model) is either an ideal surface, including location, orientation and size defects, or a skeleton model. During specification, transformation between different geometric models might be required. These transformations are ruled by an operator consisting of a set of GPS basic operations such as partition, extraction, filtration, association, reduction, etc.

#### 2.3.2. Specification Feature Model

**Figure 4:**
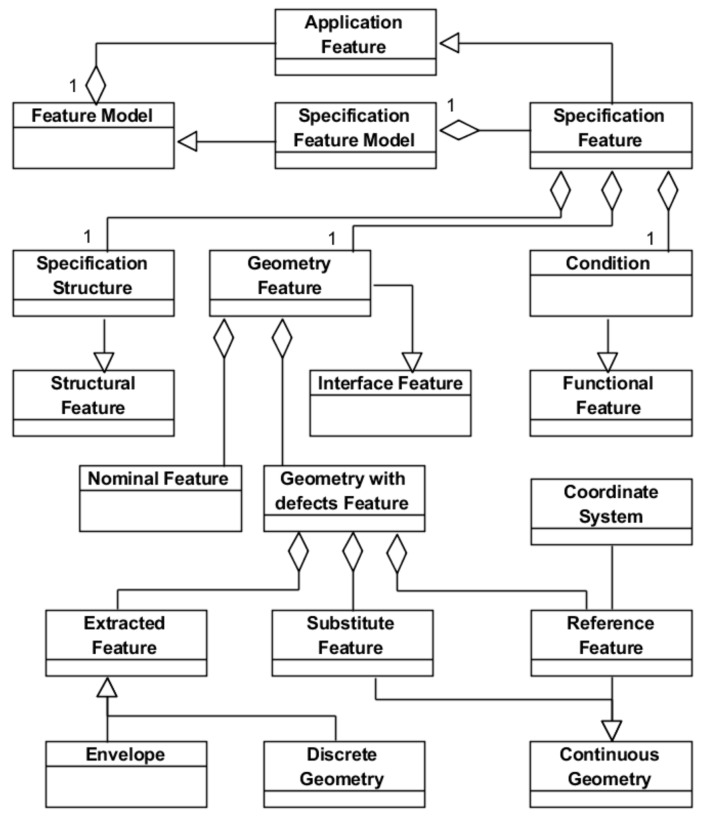
Specification Feature Model (updated from [13]).

The three specification exercises (product, manufacturing plan and inspection plan) involve the management of geometrical variability, although different names are used in each specification field: tolerance in product design, natural process tolerance (capability) in manufacturing and uncertainty in inspection. For that reason, the three specification exercises should be based on a unified feature model where the geometrical interface is represented using the same geometry model as described in the previous section. Based on this assumption, authors of this work proposed a unified Specification Feature (Figure 4) that will be briefly summarized in this section [13]. This feature aggregates the three types of object feature considered in any Application Feature: *Geometry Feature* (geometric interface), *Specification Structure* (structural elements) and *Condition* (functional geometrical condition for which the structural elements are a solution).

As Figure 4 shows, that the geometric interface contains, the nominal geometry (*Nominal Feature*) and additionally the representation of the deviations for this geometry (*Geometry with defects Feature*) in any of the tolerance models (GPS, TTRS, etc.) necessary to support the corresponding specification exercise. The *Geometry with defects Feature* aggregates three features: (1) *Extracted Feature*, which represents the geometry in the form in which it is extracted from part surfaces with defects; (2) *Substitute Feature*, which represents an ideal and continuous geometry related to the geometry with defects; and (3) *Reference Feature*, commonly known as Datum Feature, which represents an ideal geometry that positions extracted and substitution geometries. An *Extracted Feature* can be of two types: *Discrete Geometry* or *Envelope*. When it is a discrete geometry, it is made up of a set of points, segments or tessellation elements. Otherwise, if the extracted geometry is of type envelope, it is made up of a set of (two) trimmed ideal and continuous lines or surfaces enveloping, internally and/or externally, the real geometry with defects. This second type of extracted geometry is not considered by the GPS standard, where only extracted models able to support the way in which coordinate measurement machines take measures are considered.

#### 2.3.3. Assembly Model for Specification

**Figure 5:**
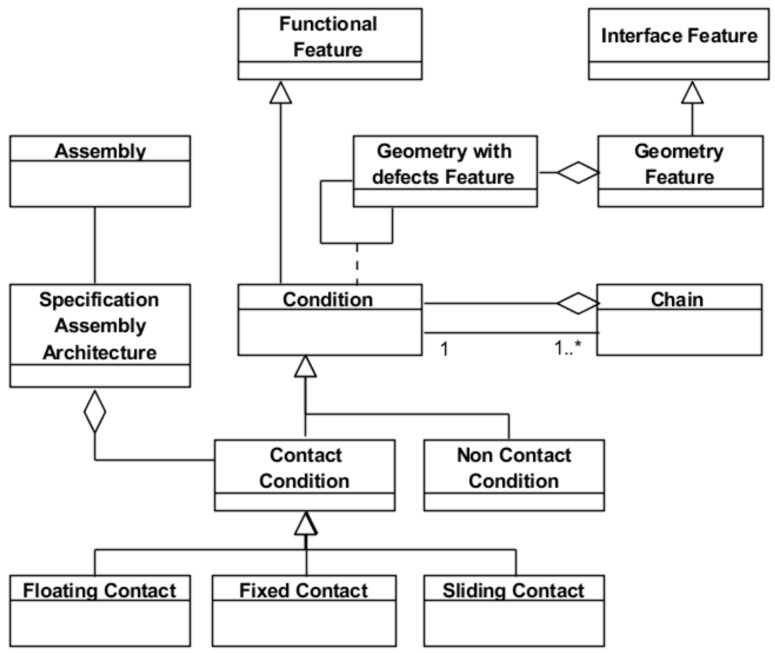
Assembly Model for Specification (updated from [13]).

The geometry with defects (*Geometry with defects Feature*) of the Specification Feature, seen in Figure 5, is the central element of the Assembly Model for Specification. This is a key model in order to establish conditions on kinematic loops associated with a mechanical assembly (product or process simulation exercises). These loops are determined according to the different assembly configurations established by the set of joints between the geometric interfaces of the different components. Therefore, for the specification exercise a model including both the assembly architecture and the chains and functional conditions is required. The links in these loops establish the relationships between the geometric interfaces that may belong to the same or to different parts. These interfaces will be represented by the corresponding geometry with defects included in the Specification Feature previously described.

The model establishes the relationships between this geometry with defects and other concepts involved in the simulation exercises, such as the specification architecture and loops (*Specification Assembly Architecture* and *Chain*). In particular, an assembly is characterised by an architecture defined as an aggregation of all contact conditions (*Contact Condition*) between the geometry with defects of all features, either in the product or in the process assembly. The types of contacts considered in the model are *Floating*, *Fixed* and *Sliding Contact* [29]. The model also considers the non-contact conditions (*Non-Contact Condition*) that establish either a condition within the same part or a separation condition between two different parts. In addition, a *Chain* aggregates all the associations between the features including the information about the geometry with defects required to close the functional loop. From all links included in the *Chain*, just one of them is associated with the condition (*Condition*) to be fulfilled (either product or process condition), and the rest of the links will be associated with other conditions (contact or non-contact).

### 2.4. Methodolgy

The methodology used to develop and validate at a conceptual level the proposal of the Inspection Feature can be summarized as follows:

1. Development of a functional model for inspection process planning in an integrated product and process (machining an inspection) development context, especially fostering in-line inspection. In this way, part quality inspection plans can feed product quality assurance and the resulting activation of management strategies. These strategies allow for smooth defect propagation throughout the process stages and to the final customer. The functional model, developed using IDEF0, enables to identify the main information requirements and shows at the aggregate level the relationships between the tasks involved in inspection process planning, machining process planning and product design. Furthermore, in order to ease the integration of all these planning tasks, a dual activity model for both process planning tasks is established. This activity model is supported by a common part representation based on a single feature concept, the Specification Feature.
2. Study of the following topics:
  - Tolerance information models used in CAT (Computer-Aided Tolerancing) applications, both for the interpretation models (such as vector equation model, variational surface model, kinematic model, degree of freedom (DOF) model, etc.) and for the representation models (such as surface graph model, technologically and topologically related surface (TTRS) models, category theory model, GeoSpelling model, ontology-based model, etc.). In particular, the concepts considered by the Geospelling language and the GPS standard are revisited.
  - Measurement processes and systems. More particularly, the ways in which the part can be situated (oriented and/or located) in relation to the geometries of the measurement resource are studied. Additionally, the alignment operations, either physical or by means of calculations (verification operator), that are performed during the verification process, are also analyzed.
  - The role of tolerancing in the context of the uncertainty management, in order to ensure that the product meets its functional requirements.
3. Development of a proposal for the specification exercises carried out in inspection process planning, which is dual to the one established for the specification exercises in product design. Accordingly, inspection plan specification (including analysis and validation) is addressed using similar assembly models, geometry models, which incorporate the representation of defects, and tools and techniques for variability management.
4. Analysis of the general UAF framework and Specification Feature Model to determine their suitability to provide a specific solution for inspection planning.
5. Development of an Inspection Feature Model and an Inspection Assembly Model based on the general UAF framework and Specification Feature Model. In particular, the models for the inspection planning domain should be adequate to support the definition, analysis and validation of the set-ups included in the inspection plan and the allocation of the inspection resources.
6. Categorization of Measurement Resources in generic types that include all type of measurement equipment, ranging from basic instruments to coordinate-based machines. The generic types of Measurement Resources have been established based on the degrees of freedom characterising the movement axes of the inspection equipment and the axes including sensors to register measurement data.
7. Development of an Inspection Feature Library. The library classes are based on the study, from measurement viewpoint, of the different geometry types that can be present in mechanical parts. The definition of the different types of features considers the way each type of feature interacts with the resource interface features corresponding to the defined Categories of Measurement Resources. The knowledge about compatibility between the part and resource interfaces is essential for the inspection planner in order to allocate the most appropriate resource. This knowledge is embedded in the form of compatibility constraints and properties of interaction
8. Validation of the proposed models by the application to several case studies. The aim is just to validate that the concepts supporting the Inspection Feature Model are adequate to select and analyze an inspection solution. A developed graph-based methodology that supports the inspection chains representation corresponding to each characteristic to be verified in one set-up is used in order to facilitate analysis and validation exercises of the inspection solution.

In this work, only stages 3, 4 and 5 are covered due to space limitations, although a simple case study is presented so that the reader can see how the Inspection Feature can support the involved tasks in the inspection process plan specification. The rest of the stages are out of the scope of this work and they will be the object of future publications.

It should be noted that author’s interest does not aim the development of an object-oriented application for inspection planning. Therefore, although UML notation has been used to show the concepts of the proposed model, the UML classes have not been detailed with their attributes and operations.

## 3. Results: Feature-Based Framework for Inspection

As already mentioned, product specification involves:

- The definition of an assembly (product artefact) that can be a technical solution for the required functionality expressed as functional conditions. The technical solution is a set of parts with their particular geometrical shapes that are kinematically related through their geometrical interfaces.
- Since the part geometrical interfaces will have defects (intrinsic or extrinsic), different characteristics limiting them have to be specified. A specified characteristic is a characteristic with the permissible (maximum and minimum) limits, where a characteristic is a linear or angular distance defined between geometric elements (ideal or non-ideal) [27]. Each specified characteristic requires the definition of a GPS operator that establishes the procedure to obtain it from the data of the involved geometrical elements.
- The validation whether the total assembly performance (tolerance) meets the functional condition. This is calculated through a chain that considers the characteristics of the assembly components (individual parts) and the contact conditions.

Similarly, for each specification to be verified, the inspection plan specification involves:

- The definition of an assembly (inspection artefact), formed by the subject part of the inspection and a set of components (measurement resource, fixture, probe, etc.). This assembly must be a technical solution capable of extracting part geometric information needed for the verification of the specified characteristic.
- Since the extracted part geometry will have defects, the planner, similarly to the designer, uses ideal geometry models that enable him/her to represent the measured geometry with defects. Working on these imaginary ideal geometries with defects, which belong to both the part and the rest of the assembly components (measurement resource, fixture, probe, etc.), the planner establishes the GPS operator to obtain characteristics to be measured corresponding to the specified characteristics.
- The validation whether the total uncertainty (method and implementation) of the inspection assembly meets the requirements of the verification of the specified characteristic. This uncertainty is calculated through a chain that considers the characteristics of the assembly components (part and inspection resource) and contact conditions.

As is clear, in product specification and inspection plan specification very similar tasks must be undertaken. Furthermore, both specification exercises work on a common representation of the geometry with defects of the part, either of the conceived or of the real one. GPS operators are applied to the geometry with defects in order to quantify the characteristics and their variability. When the operators used for both exercises are coincident (duality), then uncertainty is minimised.

Hence, the specification of the inspection process plan involves establishing GPS operators on the verification geometries of both the part and the components of the inspection resource and analyzing the contacts between the previous geometries. Therefore, in the first part of this section the specific geometries needed for verification (part and inspection resource) are going to be studied. In the next two parts of this section, an inspection feature and assembly models are proposed. These models are based on the verification geometries. Finally, a case study is presented.

### 3.1. Geometry Model for Verification

**Figure 6:**
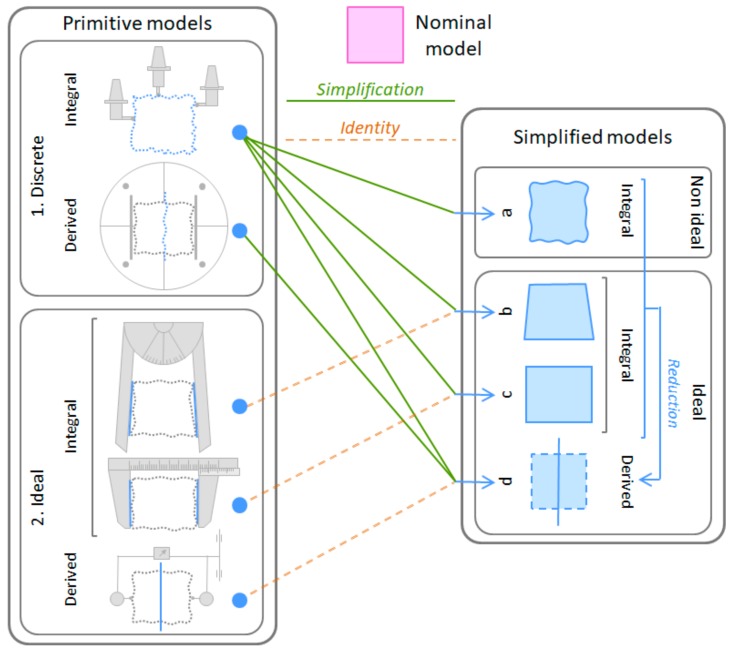
Part geometry models for verification.

Usually, in product specification the designer considers skin and/or skeleton models with defects for a GD&T analysis process based on simulation [29]. These models are constructed from the nominal model based on ideal geometries with imagined dimensional and angular defects. As shown in Figure 6, these imagined geometries with defects are represented by the Substitute and Extracted Features defined in relation to a reference geometry represented by the Reference Feature, which usually is the same as the Nominal Feature.

However, in the specification of the inspection plan the planner considers skin and/or skeleton models with defects imagined as a result of the extraction process. The type of these imagined extracted geometries depends on the type of extracted geometry that the inspection resource can provide.

**The Geometry of the Part**

In order to verify a part characteristic, the inspection planner must have an adequate representation of the part real geometry. This representation should enable the planner to establish the verification GPS operator, as a set of several GPS operations (partition, extraction, association, etc.). This GPS operator will include a last evaluation operation to allow for the verification of the characteristic. The representation of the part geometry, gathered during the measurement process, is referred in this work as “verification primitive model”. Hence, the GPS operator established by the planner will operate on this verification primitive model transforming it into simpler ones from which the required linear and angular distances to complete the evaluation operation of the characteristic can be computed.

The verification primitive model for inspection plan specification, unlike product specification, is very often a discrete model, obtained by sampling a finite number of points, segments or tessellation elements on real part surfaces. The vast majority of specialised literature, including the GPS standard, assume that inspection plan specification starts with this type of verification primitive model (discrete model).

However, this situation in only present when metrological systems based on coordinate measuring processes equipped with (mechanical and optical) probes are used. When basic metrology, such as a calliper, is employed, the verification primitive model is a much simpler one, since from the available information only an ideal profile model with dimensional defects (due to linear and/or angular variations) can be obtained. As Figure 6 shows, this profile model does not consider part form and orientation defects, since these are neglected by the contact surfaces of the inspection resource assumed geometrically perfect.

Then, the verification primitive model is a representation of a real instance of the part geometry that depends on the extraction method and the inspection resource used, and can be of two main different types (Figure 6, left):

1. Discrete models with defects (integral or derived profiles/surfaces) consisting of sets of points, segments or tessellation elements (with a particular pattern). These models are obtained when measurement is performed by equipment that provides coordinate information, such as CMM (Coordinate Measurement Machine), optical equipment, surface form/texture metrology, etc. The coordinate information is referred to the equipment coordinate system that is realised by the movements of its guideways. To make this equipment very flexible, its guideways can be linear, resulting in rectangular coordinate systems, or a combination of linear and angular movements, resulting in spherical, cylindrical, etc., coordinate systems. There is equipment with two guideways that can be used to obtain two dimensional discrete models and others with three or more guideways that can be used when three dimensional discrete models are required.
2. Ideal models with dimensional defects that keep the nominal form. These models are obtained when measurement is performed either by conventional equipment (calliper, micrometer, goniometer, etc.) or by equipment and set-ups used in comparison measurements. The first ones provide a specific linear or angular distance between two ideal geometries that are embodied by the measurement equipment. The second ones provide two linear or angular distances (maximum and minimum deviations) that enable the construction of two ideal geometries (surfaces or profiles) that are internally or externally enveloping the real part geometry. The construction of these two ideal enveloping geometries is performed by the movement of the measurement equipment guideways (sweeping movement). When surface models (3D) are required, it will be necessary to use two axes for the sweeping movement (two isoparametric lines), resulting in an enveloping surface. However, if plane profile models (2D) are desired, just one axis for the sweeping movement will be required, resulting in an enveloping line. When surface models of a complete partitioned geometry using any of these two types of measurement processes (conventional equipment or set-ups for comparison measurements) are desired, measurements in several planes (parallel, coaxial, etc.) will be required in order to cover the whole partitioned surface. Obviously, the uncertainty of these surface models with dimensional defects will depend on the possibility of coincidence of the reference geometry with these profiles, as it will be explained in the next section.

From these primitive models, the verification GPS operator can establish other simplified models (Figure 6, right) required to assess the part specified characteristics. In particular, the verification operator can establish the following simplified continuous surface/profile geometric models with defects: (a) Non-ideal models, which are generated by reconstruction operations (fitting and interpolation) from the primitive models with the aim of obtaining the points that match with the sampling points established in the specification; (b) Ideal models with angular defects; (c) Ideal models with linear dimensional defects; and (d) Ideal derived models that can be obtained either by a GPS derivation operation from the previous simplified models or directly from a derived primitive model resulting from a measurement process.

Figure 6 also shows that when the primitive models are ideal, they are the same as the corresponding simplified models (b–d). Although it is often unnoticed, very often the primitive geometry itself already contains information (measurements) about the specified characteristics and, therefore, no subsequent transformation of the geometry will be necessary to obtain these characteristics. This is the case of many dimensional characteristics associated with a specific geometrical element that are obtained by direct measurements of dimensions (angle, diameter, width, etc.) or by sweeping processes. When using sweeping processes, the measurement process or equipment does not register deviations of specific points of the geometrical element of the part, and provides only the total deviation produced in the sweeping process of specific profiles or of the complete surface. A classic example of this type is the measurement of the straightness of a plane using a rule and an indicator.

**The Geometry of the Inspection Resource**

In addition to the real part geometry, in verification, an inspection resource is also involved in the measurement process. This inspection resource has real geometries of high quality that are always assumed to be ideal, neglecting their defects, since they are usually very small. Examples of these geometries are the surface of a plate, the axis of a chuck, etc. Based on this assumption, the real geometries of the inspection resource are represented using ideal models (without defects) for all the reasoning and computing processes required to obtain the measured characteristic. The uncertainty of the measured characteristic is influenced by the quality of the real geometries assumed as perfect.

Generally, in order to obtain a measured characteristic between two geometries (target and datum) the comparison of the real target geometry in relation to the datum frame geometry (specification reference geometry) is required. This comparison involves obtaining linear and angular distances in relation to this specification reference geometry. In turn, this specification reference geometry can also be considered as a target geometry, whose measurement involves comparing it with another datum frame geometry (measurement reference geometry).

Therefore, every measurement reference geometry can be used as a specification reference geometry. The measurement reference geometry, in relation to which linear and angular distances are obtained, is always realised by the inspection resource. This realization, as it will be explained later in this section, can be of different types, such as a flat surface contact, an axis of a revolved surface, etc. On the other hand, the specification reference geometry, which is always required for the measurement of a specified characteristic, is obtained either by a measurement process comparing it with a measurement reference geometry, or by doing it coincident with a measurement reference geometry embodied by the inspection resource using an alignment process.

**Figure 7:**
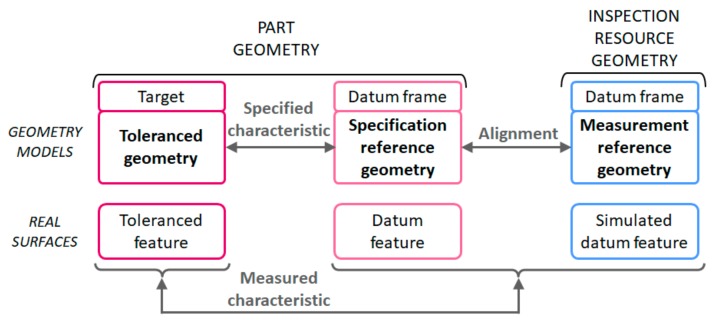
Geometries involved in verification.

In general, these three geometries (tolerance geometry, specification reference geometry and measurement reference geometry) are involved in the measurement of a specified characteristic (Figure 7). According to the specified characteristics and the selected measurement process, some of these geometries are the same. For example, when form characteristics are verified, the specification reference and the target reference can be the same. When orientation and location characteristics are verified, the specification reference and the measurement reference can be the same.

The real geometries of the inspection resource considered as ideal models are normally known as embodiments in the metrological domain. The linear or angular distance values obtained by the inspection resource are always referred to these embodiments that are the reference for the measurements. Embodiments to establish the measurement reference can be also other ideal geometries that are of the same type to the previous ones (real geometries of the inspection resource). They are usually an offset of the real ones and are established during the equipment set-up process. For example, when a parallelism specification is inspected by means of a set-up using a surface plate, a height gage and a dial indicator, the reference measurement can be the surface plate itself contacting the specification reference. However, an imaginary plane parallel to the surface plate with a specific offset controlled by the height gage could also be used as the measurement reference.

The embodiment of the “measurement references” by the inspection resource can be of one of the following types:

- Positioning embodiment, when the reference is realised by physical contact with surfaces of the equipment or set-up (e.g., gusset plates, mandrel, precision jaws, precision fixture, etc.) or is realised as an offset of the previous ones by gauges used during the setting or calibration process of the equipment.
- Kinematic embodiment, when the reference is realised by the movement of the measurement equipment guideways. Obviously, this reference is located in the inspection resource, since the guideways used to generate it have a specific location in the equipment. The number of measurement equipment guideways has to provide the minimum number of independent axes required by the type of tolerance geometry.
- Calculated embodiment, when the reference is obtained by mathematical association operations using the part extracted points, segments or tessellation elements and appropriate criteria such as least square, minimum outer diameter, etc.

Not all types of references embodied by the inspection resource can be used with all types of primitive models of the tolerance geometry. In particular, the reference as calculated embodiment (c) leads to discrete part primitive models of the tolerance geometry, which can be simplified to ideal geometries if appropriate. On the other hand, kinematic embodiments (b) or positioning embodiments (a) lead to an ideal part primitive model of the tolerance geometry. More specifically, the ideal part primitive model obtained using a kinematic embodiment is a set of two ideal geometries enveloping the real geometry. These two ideal enveloping geometries are of the same type and are generated simultaneously with the kinematic embodiment geometry. However, the positioning embodiment leads to an ideal part primitive model that is an ideal geometry establishing a single boundary (external or internal) of the real geometry.

As has been mentioned, the measurement reference is embodied by the inspection resource, whereas the tolerance geometry to be extracted exists on the part. In addition, to obtain the measured characteristic a specification reference also existing on the part is required. This specification reference must be located (usually by coincidence) in relation to the reference embodied by the inspection resource. This is the so-called alignment process that always introduces an additional uncertainty in the inspection process. If a misalignment between the real geometry of the part and the reference geometry appears, a misalignment error is also present.

The aim of the alignment process is basically to make two geometries, one of the parts and one of the inspection resources, coincident (orientation and situation). The measurement reference geometry is realised by real geometries of the resource (high precision surface plates, gusset plates, mandrels, etc.) known as simulated datum. The defects of these real geometries of the resource are neglected compared to the part geometry defects and, therefore, they are considered to be ideal geometries. It must be noticed that the effect of this assumption is included in the resource uncertainty obtained during the calibration process. The lower the quality of the real geometries of the inspection resource, the higher the measurement (implementation) uncertainty. The part geometry must contact with these real (assumed ideal) geometries of the inspection resource. However, since part geometry is not ideal, there is no one single stable solution for the contact. Due to the significant effect of this circumstance on the uncertainty, the use of some requirements to rule the relative location is required, such as the minimum requirement or the minimum rock requirement [30].

Very often, the alignment process is realised locating the part by physical contacts with the inspection resource minimising the deviations between part and inspection resource geometries. In these alignments by physical contact, two cases can be distinguished depending on whether the part contact surface is the same or not as the specification reference. An example of the first case is when a part flat surface directly contacts with the surface plate that orients the part and is used as reference. An example of the second case is the clamping process of a cylindrical part using a roundness measuring instrument where dial indicator values on the cylindrical surface when turning the part around the equipment axis are minimised.

In coordinate-based measurement processes, the alignment process is the calculation of the measurement reference. In this case, the alignment process involves calculating an ideal geometry that is used as specification/measurement reference and probing on its normal direction.

### 3.2. Inspection Feature Model

In this section, and based on the concepts related to the geometries with defects explained in the previous section, an Inspection Feature Model is proposed and described using UML diagrams. The Inspection Feature is defined as a subtype of the Application Feature considered in the UAF framework outlined in section.

**Figure 8:**
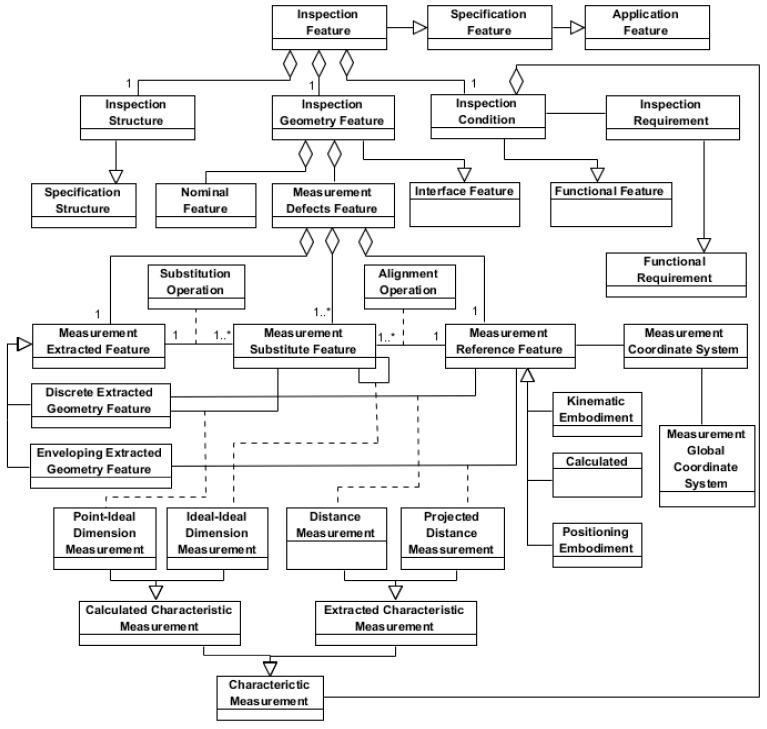
Inspection Feature (InspF) Model.

The *Inspection Feature* (InspF) shown in Figure 8, as subclass of the *Specification Feature* class, is an aggregation of the classes with the information about the structure (*Inspection Structure*), the geometric interface (*Inspection Geometry Feature*) and the functional geometric condition (*Inspection Condition*), which are established as requirements on the characteristics to be measured (*Characteristic Measurement*) in order to obtain the values of the specified characteristics (*Inspection Requirement*) that points to the self-geometries of the InspF. These specified characteristics have been established along the product design stating their GPS specification operators and their variation limits (tolerances).

The inspection process plan specification starts analyzing those specified characteristics in order to define the part geometry using the feature types from the InspF Library (feature recognition) and to establish the *Inspection Condition*. The part recognition using the InspF Library developed as stated in the methodology section, is essential to ensure: (1) that is possible to extract the measurement data for the specified characteristic calculation and (2) that there exists inspection resource type able to execute the data extraction. These inspection resource types facilitate the selection of one or more technical solutions to carry out the InspF measurement.

In the same way that *Inspection Condition* relates the InspF with the product functional structure, the *Inspection Structure* relates the InspF, and more specifically its *Nominal Feature*, with the component structure of the planned inspection assembly, in which part participates. For this, the *Inspection Structure* contains the topological structure of the InspF and positions it in the part framework.

The *Inspection Geometry Feature* aggregates two feature: (1) The *Nominal Feature*, which represents the nominal geometries of the feature that are defined as ideal geometries; and (2) The *Measurement Defects Feature*, which is used to represent the real geometries participating in the measurement process as ideal geometries that model form and location (orientation and situation) defects. The *Measurement Defects Feature* aggregates three features: (1) The primitive geometries extracted in the measurement process (*Measurement Extracted Feature*); (2) The reference geometries (datum frame) used to obtain the previous ones (*Measurement Reference Feature*); and (3) The required geometries resulting from simplification processes applied on the primitive geometries (*Measurement Substitute Feature*).

The extracted geometry with defects (*Measurement Extracted Feature*), which is a representation of the real geometry obtained as described in Section 3.1, can correspond to discrete primitive models (*Discrete Extracted Geometry Feature*) or to ideal primitive models (*Enveloping Extracted Geometry Feature*) as an envelope model, consisting of one or two ideal geometries limiting the real one.

As it has been previously explained in Section 3.1, the *Measurement Reference Feature*, which is the reference for the measured values, can be a *Positioning*, a *Kinematic* or a *Calculated Embodiment*. The *Measurement Reference Feature* can be any of the invariance classes geometries [27,29,31,32].

As Figure 8 shows, the *Inspection Condition* aggregates the characteristics to be measured (*Characteristic Measurement*). The *Characteristic Measurement* is an associative class that, in general, characterises the relation between *Measurement Defects Features*. This characterization is expressed according to Geospelling language as a set of sequenced GPS operations to establish and obtain the value of a characteristic (linear or angular distance) between any of the three components of the *Measurement Defects Feature* (Extracted, Substitute and/or Reference). The *Characteristic Measurements* can be of two main types: (a) *Extracted Characteristic Measurement*, which are characteristics between a *Measurement Extracted Feature* and a *Measurement Reference Feature* directly obtained by the inspection resource as linear or angular distances; and (b) *Calculated Characteristic Measurement*, which are characteristics between a *Measurement Substitute Feature* and either another *Measurement Substitute Feature* or a *Measurement Extracted Feature* obtained as linear or angular distances after applying mathematical/geometrical operations to values given by the inspection resource. The second type (b) of characteristic measurements are the most common ones when using inspection resources that provide a big amount of part geometrical data, such as the widely used coordinate-based measurement equipment. The latest standard developments in this field mainly focus on this type of measurement equipment.

Two types of *Extracted Characteristic Measurements* can be distinguished:

- The *Distance Measurement* is the relation between an extracted discrete geometry (*Discrete Extracted Geometry Feature*) and a reference geometry (*Measurement Reference Feature*). For example, the measurement of a distance between a point and a plane.
- The *Projected Distance Measurement* is the relation between an extracted enveloping geometry (*Enveloping Extracted Geometry Feature*) and a reference geometry (*Measurement Reference Feature*). For example, the measurement of an angular distance using a goniometer where both instrument probes contact part surfaces resulting in two ideal geometries (straight lines) whose included angle is the characteristic measurement. Both ideal geometries are enveloping extracted geometries (only one limit in this case) from the part and result in two substitute geometries (*Measurement Substitute Feature*) through an operator (*Measurement Substitution Feature*) that in this case is as simple as the identity. It must be noticed that one of them is used as the measurement reference (*Measurement Reference Feature*) being the alignment (*Alignment*) in this case the identity. Obviously, the type of reference in this case is established by the part-instrument contact (*Positioning Embodiment*). In the case of enveloping geometries with two limits, these will have the same form and location than the reference surface used for the measurement and are obtained by a sweeping process on that reference surface. This sweeping process is performed using the measurement equipment guideways.

Similarly, two types of *Specified Characteristic Measurements* can be distinguished:

- The *Point-Ideal Dimension Measurement* establishes the relation between an extracted discrete geometry (*Discrete Extracted Geometry Feature*) and a substitute geometry (*Measurement Substitute Feature*) as a sequence of GPS operations that results in the quantification of the characteristic to be verified. For example, when for the verification of a parallelism between two planes a surface plate and a height gauge are used. In this case, one of the planes contacts the surface plate (*Positioning Embodiment*) establishing the measurement reference (*Measurement Reference Feature*) and the ideal substitute geometry (*Measurement Substitute Feature*) by an alignment (*Alignment*) that is the identity. The other plane is sampled with the height gauge obtaining a discrete geometry (*Discrete Extracted Geometry Feature*) as a set of points. The GPS operator to verify the specified characteristic is the result of the difference between the maximum and minimum height (measured from the reference plane) of the set of sampled points.
- The *Ideal-Ideal Dimension Measurement* establishes the relation between two ideal substitute geometries (*Measurement Substitute Feature*) as a sequence of GPS operations between those ideal geometries resulting in the quantification of the characteristic to be verified. For example, using the previous example of a parallelism specification between two planes, but now using a CMM. In this case, both planes are sampled as a set of points in relation to the same reference measurement (*Measurement Reference Feature*). From the extracted geometry of both planes (*Discrete Extracted Geometry Feature*) the corresponding ideal substitute geometry (*Measurement Substitute Feature*) is obtained by an appropriate substitution operator (*Measurement Substitution Feature*). Between the two ideal substitute geometries, a GPS operator containing basically construction and evaluation operations is used to quantify the specified characteristic.

As has just been described, a key entity of the *Inspection Feature* is the *Measurement Defects Feature* that represents the real geometry of the part with defects through a combination of three geometries: the *Measurement Extracted Feature* and *Measurement Substituted Feature*, representing the defects on the part, and the *Measurement Reference Feature*, required in every measurement process for verification in order to orient and/or locate the first two. In addition, the model includes several associative classes to characterise, through GPS operators, the relationships between these three geometries, either for simplification and alignment purposes (*Substitution Operation* and *Alignment Operation*) or for the evaluation of the characteristic to be verified (all subtypes of *Characteristic Measurement*). The latter is related to the *Inspection Condition*, which is also included in the *Inspection Feature*.

### 3.3. Inspection Assembly

As mentioned in Section 2, the inspection planner task for the verification of a specific characteristic consists of defining an assembly, made up of the part and the inspection resource. This assembly must be able to extract the part geometric information required for the evaluation of the characteristic by a GPS operator. In addition, the planner must also validate that total uncertainty of the selected assembly is adequate for the limit established for the inspection condition. The extraction of the part geometric information, as it has been explained in the former section, involves the selection of reference surfaces in relation to which deviations, as linear or angular distances, are measured. On the other hand, the use of dual verification and specification operators will reduce the uncertainty.

The complete inspection process plan specification will include all the assemblies required to measure the InspF involved in the verification of all specified characteristics of the part. Obviously, in order to optimise the inspection process, the number of assemblies used should be minimised. Each assembly will require a set-up including the orientation and location of the part in the inspection resource, which has been previously referred as the alignment process. This alignment process can be more or less time consuming depending on the type of inspection resource and alignment and will have an influence on the uncertainty.

As previously mentioned, the established inspection assemblies are made up of two components (the part and the inspection resource) and two interactions exist between them. Each interaction includes all associative classes that describe the relations between part and resource features. The two interaction types are: (a) the location interaction, which holds, orients and/or positions the part in relation to the equipment reference system; and (b) the measurement interaction, which generates the stimulus, by contact or without contact between the probe and the part, for the registration of the sensors signals. Although the inspection resource or equipment itself is a mechanical assembly made up of several components and their interfaces, it will be considered as a whole (black box), characterized by a global uncertainty accompanying all the values of measurements carried out using that resource.

**Figure 9:**
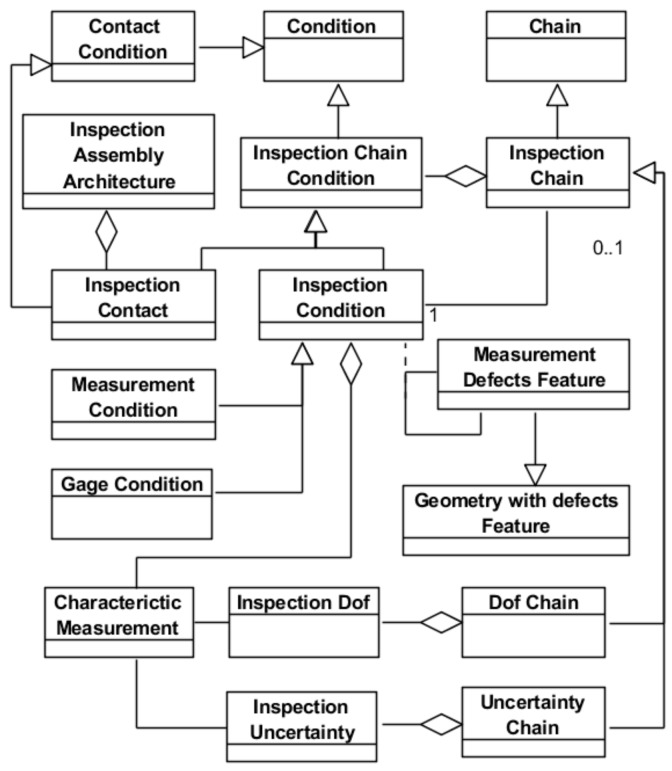
Inspection Assembly Chain Model.

Figure 9 shows the model for the inspection assembly that enables the planner to analyze and specify the inspection process by reasoning on the assembly chains or loops (*Inspection Chain*). An *Inspection Chain* aggregates *Inspection Contacts*, which represent all the fixed location interactions between the part and the inspection resource defining the assembly architecture, and *Inspection Conditions*, that aggregate one or more *Characteristic Measurement*. Each *Inspection Chain* is useful to analyze one of the *Inspection Condition* that corresponds to an *Inspection Requirement*. Usually an *Inspection Condition* is related to measurement operations that results in measurement data. This type of *Inspection Condition* is a *Measurement Condition*. However, when the part is inspected using gages, only the conformance is checked, but no measurement data is available. This type of *Inspection Condition* is a *Gage Condition*.

These chains allow for the planner the establishment and validation of the final solution through the analysis of the required D.o.f. (*Dof Chain*) and the uncertainties (*Uncertainty Chain*) introduced by the different involved elements. The uncertainty chain includes the uncertainties (*Inspection Uncertainty*) of all the relationships between geometrical measurement defects features of the part and of the inspection resource as a whole that have to be stacked up to fulfil the inspection condition. The D.o.f. chain includes the information about the required active and inactive D.o.f. (*Inspection Dof*) for part location, sweeping and measurement.

### 3.4. Case-Study

**Figure 10:**
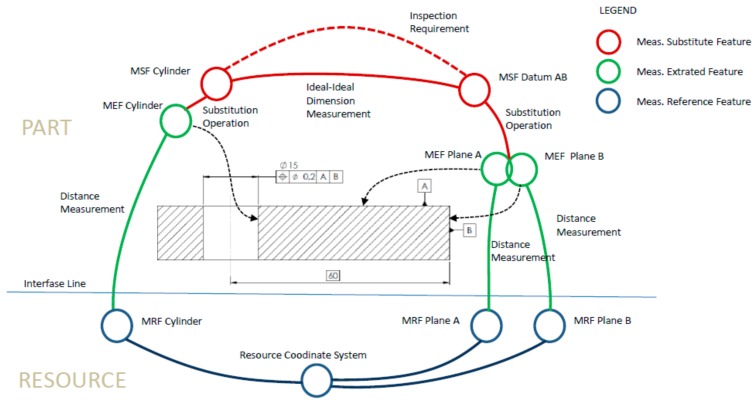
Graph including the InspF for the case-study.

In this subsection, a simple case study is described with the aim of showing how the proposed Inspection Feature Model supports the reasoning carried out in some of the tasks typical of the inspection process planning. The example considers a very simple part (see central part of Figure 10) with just one key characteristic. This characteristic has been established using a standard position tolerance specification that restricts the deviation of the hole axis in relation to a datum defined by plane A and plane B.

The specification of the process plan begins with the recognition of the toleranced geometry (cylinder and planes A and B) based on the InspF types established in the Library. In this case, the planner identifies the hole surface as one Cylinder InspF type and the two plane surfaces as two Plane InspF type. Additionally, taking the tolerance of the specified characteristic (0.2 mm) as basis, the planner establishes the Inspection Requirement with the statement: “To measure the deviation of the hole position with a maximum uncertainty of 0.03 mm”. This uncertainty value complies with the 1/6 relation usually established between the specified tolerance and the uncertainty of the measurement process.

Once the functional requirement (Inspection Requirement) has been established, the planner must find a solution to measure the characteristic. Previously, however, he/she will have to define the Measurement Substitute Features (MSF) that are capable of obtaining and evaluating the measurement of position characteristic by the application of the required construction, calculation and evaluation GPS operations. In this case, the MSF defined are two, one corresponding to the Cylinder InspF and another one aggregating the two Planes InspF of the compound datum AB. When these MSF have been determined and taking into consideration the requirements compelled by the InspF types they belong to, different inspection solutions can be examined.

For this, several alternative *Measurement Reference Features* (MRF) for each MSF can be considered. Next, for each of these alternatives, a series of requirements must be established on the *Characteristic Measurements* necessary to fulfil the Inspection Requirement. These *Characteristic Measurements*, which constitute the *Inspection Condition*, are established in terms of uncertainties and D.o.f.

In particular, for this case study, the MDF that could be linked to the MSF corresponding to the hole and to the datum AB could be any of the types considered in the model (*Kinematic Embodiment*, *Calculated*, *Positioning Embodiment*). However, some of these MDF would be difficult to realise and should be disregarded. Furthermore, if as usual the MDF are kinematic embodiment or calculated, the *Measurement Extracted Features* (MEF) required to obtain the MSF should also be defined.

Usually, since several alternative MRF will have been defined for each InspF, the planner should study whether an MRF corresponds to more than one InspF, because the existence of MRF common to several InspF helps to minimize the number of required inspection assemblies (set-ups). In the case at hand, given its simplicity, it is clear that the two MSF can be obtained using a single inspection assembly and the following alternative solutions could be considered:

1. Measurement of the cylinder using a resource of type “measurement on axis” (e.g., center bench). Planes A and B would be used to locate the part on the resource by means of a location gage 3–2.
2. Measurement of the cylinder and of plane B using a resource of type CMM. Plane A would be used to locate the part on the resource.
3. Measurement of the cylinder and of planes A and B using a resource of type CMM. No specific location of the part is required in this case.

Although the specification of any of the three alternatives could be object of study, only the third is going to be analyzed. The analysis will be supported by the construction of the graph shown in Figure 10. Following the previously described procedure, MSF are first placed in the graph and later the MRF and the coordinate system of the resource are also placed. In this case, as the selection of solution has already been made, only one MRF is represented for each MSF, all of them of *Calculated* type. Thus, three MEF are also incorporated in the graph. These MEF correspond to the cylinder and the two planes. Proceeding with the graph construction, the thus far represented entities are linked by lines that symbolise the relationships established among the entities. In this case, two types of relationships can be established, i.e., *Distance Measurement* and *Substitution Operation*. The whole set of links is a graphical representation of the *Inspection Chain* that supports the identification of the involved uncertainties and D.o.f. chains.

As the graph shows, there are some entities that belong to the part (placed above the interface line) and others that belong to the resource (placed underneath the interface line). It can also be noted that there are some links that cross the interface line. These links are instances of the *Extracted Characteristic Measurement* and represent the measurement interaction between the part and the resource. In this case, there are no links representing the location interaction, since part location is not involved in the measurement.

**Figure 11:**
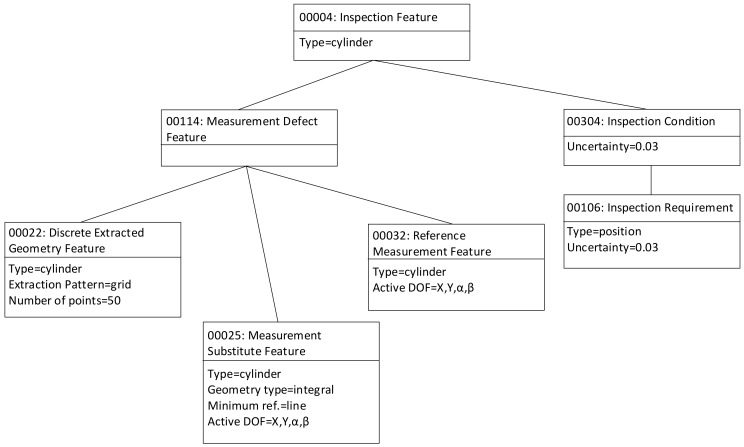
Some object instances and relations of the case study.

Finally, although the objective of the present research work is not the development of an object-oriented application for process planning, as already mentioned in the methodology subsection, Figure 11 shows some instances of the entities and relationships defined in the case study for the Cylinder InspF using a UML object diagram. The aim is to help the reader in the comprehension of the case by detailing some of the attributes of the classes of the InspF Model. As can be seen in Figure 11, among the object attributes those required to build and analyze the D.o.f. and uncertainty chains can be found.

## 4. Conclusions and Future Work

In this work, a feature-based framework for inspection has been proposed. This framework is a specialization of a more general feature-based framework that supports the specification, analysis and validation of any technical solution (artefact). In this general framework, the Application Feature plays a key role since it is an informational object that carries the mapped functional and the structural solutions.

The development of the proposed feature-based framework for inspection has enabled to prove that the general feature-based framework is adequate not only for the specification, analysis and validation of GD&T characteristics on components of product artefacts (assemblies), but also for process artefacts (assemblies), more particularly for inspection assemblies. These inspection assemblies participate in the execution of the operations included in a set-up of the inspection plan. An inspection assembly (set-up) is made of two components: the subject part of inspection and all measurement devices (chucks, rules, plates, gages, probes, guideways, etc.) that together constitute the measurement resource.

As part of the feature-based framework for inspection, the Inspection Feature (InspF) is an essential element because it contains the necessary information to check the compatibility between the part and resource features allowing, as exposed in the included case-study, the specification and validation of inspection assemblies.

The results of this research show the possibilities of the proposed Inspection Feature for the development of knowledge-based applications in the field of inspection planning. The proposed model supports the design/selection of inspection solutions in collaborative production contexts, described in the introduction. However, from a conceptual point of view, additional work to validate the proposed approach is still needed. To that end, it is proposed, on one hand, to study in depth the inspection interaction from the resource perspective, and, on the other hand, to test the model consistency by stating an ontological model implemented in OWL (Ontology Web Language) and SRWL (Semantic Web Rule Language). In addition, the ontological approach will allow the incorporation of knowledge required to support process planning tasks, enabling the automated reasoning, the capture of new knowledge through the addition of new rules, etc.
